# Activation and inactivation of *Bacillus pumilus* spores by kiloelectron volt X-ray irradiation

**DOI:** 10.1371/journal.pone.0177571

**Published:** 2017-05-11

**Authors:** Thi Mai Hoa Ha, Derrick Yong, Elizabeth Mei Yin Lee, Prathab Kumar, Yuan Kun Lee, Weibiao Zhou

**Affiliations:** 1 Singapore Institute of Manufacturing Technology, Singapore, Singapore; 2 School of Electrical and Electronic Engineering, Nanyang Technological University, Singapore, Singapore; 3 Food Science and Technology Programme, c/o Department of Chemistry, National University of Singapore, Singapore, Singapore; 4 Department of Microbiology, National University of Singapore, Singapore, Singapore; University of Connecticut, UNITED STATES

## Abstract

In this study, we investigated the inactivation efficacy of endospore-forming bacteria, *Bacillus pumilus*, irradiated by low-energy X-rays of different beam qualities. The different low-energy X-rays studied had cut-off energies of 50, 100 and 150 keV. *Bacillus pumilus* spores (in biological indicator strips) were irradiated at step doses between 6.5 to 390 Gy. The resulting bacteria populations were then quantified by a pour plate method. Results showed that X-rays of lower energies were more effective in inactivating bacterial spores. In addition, an increment in bacterial population was observed at doses below 13Gy. We attributed this increase to a radiation-induced activation of bacterial spores. Four kinetic models were then evaluated for their prediction of bacterial spore behavior under irradiation. This included: (i) first-order kinetics model; (ii) Shull model; (iii) Sapru model; and (iv) probabilistic model. From R^2^ and AIC analyses, we noted that the probabilistic model performed the best, followed by the Sapru model. We highlighted that for simplicity in curve fitting the Sapru model should be used instead of the probabilistic model. A 12-log reduction in bacterial population (corresponding to a sterility assurance level of 10^−6^ as required in the sterilization of medical devices) was computed to be achievable at doses of 1000, 1600 and 2300 Gy for the three different X-ray cut-off energies respectively. These doses are an order in magnitude lesser than that required in gamma irradiation. This highlights the applicability of cheaper and safer table-top X-ray sources for sterilization application.

## Introduction

Sterilization is a key process in medical device manufacturing and the food industry. In developed countries, approximately 40 to 50% of disposable medical products manufactured are sterilized using ionizing radiation, which includes gamma, X-ray and e-beam [1]. Ionizing radiation has been reported to cause direct damage to biological cells through the ionization of biomolecules or indirect damage by the generation of reactive oxygen species [2, 3]. The extent of damage depends on a combination of various factors that can include the type of irradiation, cell type, absorbed dose, dose rate, etc. [4, 5]. Among the different cell types, bacteria are often the targets for sterilization processes. In that regard, bacterial spores–being significantly more resilient than vegetative cells [6, 7]–are frequently used for benchmarking in sterilization studies. This resistance of bacterial spores to ionizing radiation has mainly been attributed to their compositions and DNA repair mechanisms [8]. Bacterial spores are thus widely recognized as biological indicators for evaluating sterilization equipment or validating sterilization processes [6–10].

Among the above-mentioned sterilization techniques, the use of low-energy tabletop X-ray sources for food disinfection and sterilization of medical devices (made of low density material) has lately attracted more interests. X-rays generated from these sources are of low energies, existing in the range of tens to hundreds of keV. A key merit of these small footprint sources is its low shielding requirements. This minimizes concerns of radiation protection, unlike when using gamma radiation, and thus highlights the potential of such sources for in-line sterilization processes.

Current studies on low-energy X-ray irradiation have mainly been focused on food disinfection. These studies have primarily investigated the post-irradiation physical, chemical, textural and sensory properties of foods such as dates [11], almond and walnut [12], spinach [13, 14], lettuce [15], seafood [16, 17]. They typically study common foodborne pathogens such as *Escherichia coli* O157:H7, *Cronobacter sakazakii*, *Salmonella enterica* or *Listeria monocytogenes*. In these studies, food items are irradiated using low-energy X-rays using a fixed energy spectrum.

It is generally accepted that irradiation efficacy is directly proportional to the absorbed dose, which is defined by the energy taken up per unit of mass. However, the effects of ionizing radiation not only depend on dose but also the energy spectrum of the irradiation source–described by the source’s beam quality. As explained in linear energy transfer (LET), different charged particles impart different amounts of energy to the material they traverse. Although X-rays and gamma rays do not comprise charged particles, LET is still commonly used to describe them, as energy transfer can occur via electron tracks [18]. In the case of an X-ray source with a defined inherent filter, its LET is determined by the cut-off energy of said energy spectrum. For low-energy X-rays, the photoelectric effect is dominant, leading to a higher LET. Consequently, lower energy X-rays, with corresponding higher LET, have been reported to offer a higher relative biological effect (RBE) [19, 20]–i.e. irradiation efficacy on biologics. The same relationship between LET and RBE has previously been established in detailed studies for charged particles [21, 22].

In recent reports, more studies have looked at bacterial inactivation by low-energy X-rays. These studies highlight the lower doses required in comparison to high-energy irradiation sources like gamma rays. There is however, to the best of our knowledge, no detailed report that compares the efficacy of bacterial inactivation across different low-energy X-ray beam qualities. Comparisons can only be made between studies on the same target bacteria and substrate. For example, two different groups have studied the inactivation of *Escherichia coli* O157:H7 on spinach leaves. They report D_10_ values (a 90% reduction of the initial bacterial population) of 21 Gy [23] and 1.1 kGy [24] for low-energy X-ray irradiation from different sources operated at different tube voltages of 70 kV and 150kV respectively. In these studies, a fair comparison is made even more difficult by the use of filters– 0.127mm thick beryllium window in [23] and was not stated in [24]. It thus becomes important that a study between different X-ray beam qualities from the same source be made. This is particularly relevant when designing sterilization protocols for different products using the same X-ray source.

In this work, we investigated the response of *Bacillus pumilus* spores by low-energy X-ray irradiation. In particular, we studied the effects of X-ray beam quality (determined by its energy spectrum) on the inactivation of *Bacillus pumilus* spores. This entailed the identification of ideal X-ray source parameters for sterilization processes. We further evaluated mathematical models based on their fitting of experimental results. These models were subsequently used in the prediction of reduction dose values.

## Materials and methods

### *Bacillus pumilus* spores

*Bacillus pumilus* (ATCC No. 27142) spores were used as reference standard in evaluating of gamma radiation sterilization process due to its high radiation resistance. *Bacillus pumilus* spore strips (dimension 1.5’ x 0.25’) were purchased from MesaLabs (Omaha, USA) at an initial population of approximately 10^7^ CFU g^-1^. MesaLabs reported a purity of >99.99%. These strips were kept inside a dry cabinet at 25°C and relative humidity of 50–60% prior to irradiation.

### X-ray irradiation system

**Fig 1:**
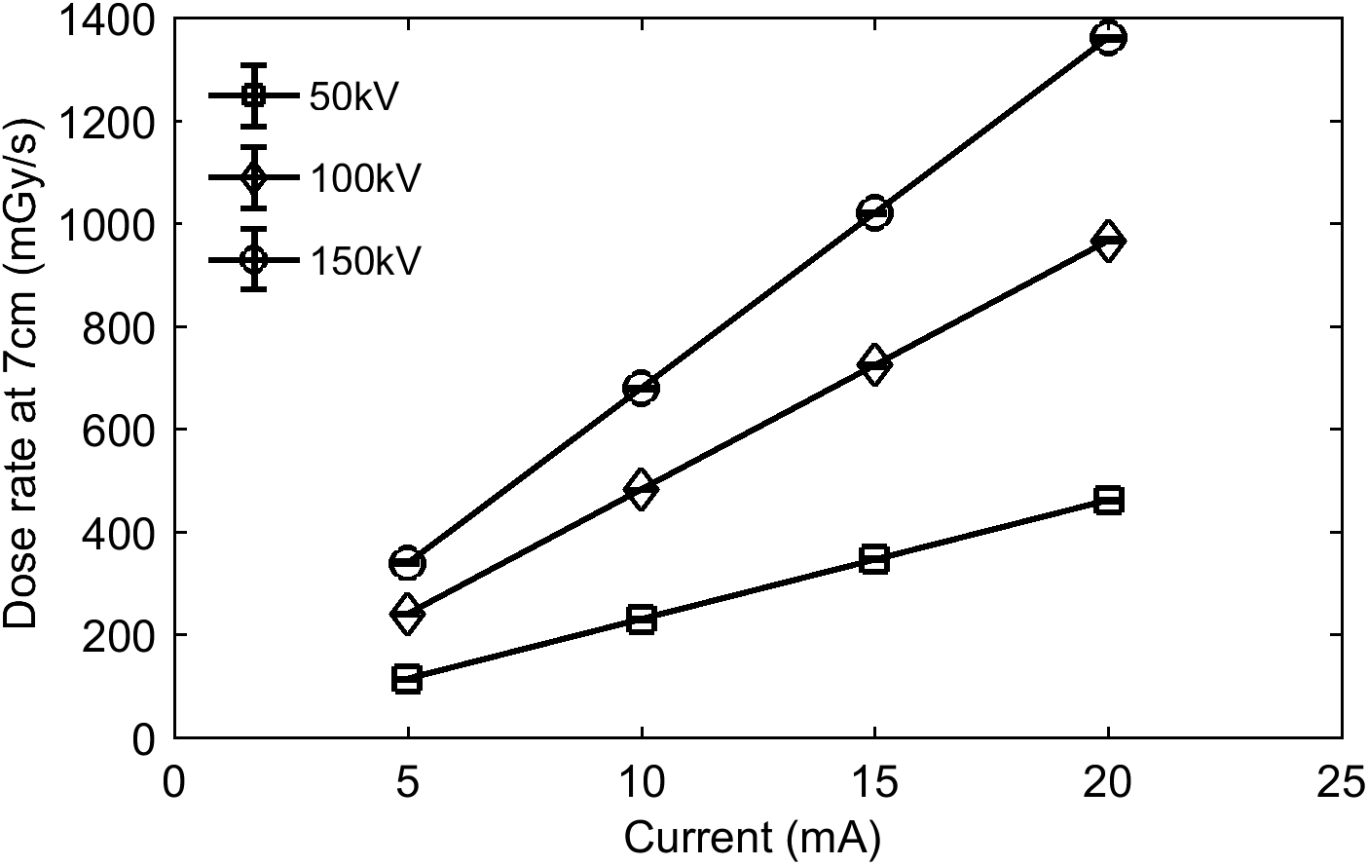
Dose rate in air measured 70 mm from the source as a function of current.

A high flux X-ray source from COMET model MXR225/26 (Flamatt, Switzerland) was used for irradiation. This source generated X-rays of up to 3 kW with a tunable broadband energy spectrum of up to 160keV bandwidth. The X-ray fluence spectrum was filtered using a 0.8 mm beryllium window. The ion chamber model 10X6-0.6 Accu Dose (Radcal, California 91016, USA) was used to measure the dose rate at a given configuration of X-ray source. Two main parameters of X-ray source that determine the dose rate at a given position are high voltage and current. The voltage (set in kilovolts or kV) applied to the X-ray source will determine the cut-off energy of the emitted X-rays (measured in kiloelectron volts or keV). Current, on the other hand, is proportional to the number of emitted X-rays, and is therefore directly proportional to the dose rate as can be seen in Fig 1.

### Irradiation of *Bacillus pumilus* spores

The X-ray source’s tube voltage was switched between three different levels (50, 100 and 150 kV) in order to generate different energy spectra. The source’s current setting was changed accordingly such that dose rate from the source was fixed at 0.65 Gy.s^-1^ in air. Biological Indicator (BI) strips containing *Bacillus pumilus* spores were exposed to X-rays at different doses (6.5, 13, 26, 52, 78, 130, 195, 260 and 390 Gy). The irradiation was performed at standard room conditions (25°C and 50–60% humidity) in triplicates.

### Enumeration of *Bacillus pumilus*

The total bacterial count was performed by the pour plate method on nutrient agar at 37°C. First, bacterial spores were extracted from the BI strips. Each BI strip was immersed in 10 ml of peptone water (product code: CM0009, Oxoid, SG) and vortexed for 20 min. 1 ml of the resulting mixture was aliquoted into a fresh 15 ml tube containing 9 ml of peptone water and thoroughly mixed. Similarly, 1 ml from this mixture was aliquoted into another fresh tube containing 9 ml of peptone water and mixed. This serial dilution was performed for a total of six times. Following dilution, 1 ml from each of the six 15 ml tubes was extracted and transferred into a petri-dish each. 15 ml of nutrient agar solution was next added to all petri-dishes. The nutrient agar solution was prepared from nutrient agar powder (product code: CM0003, Oxoid, SG) according to the manufacturer’s instructions. Immediately after adding the nutrient agar solution, the petri-dishes were rotated clockwise and anti-clockwise to evenly distribute the sample. The solutions were next allowed to cool down and solidify. Then, the petri-dishes were incubated in an inverted orientation at 37°C for 2 days. At the end of incubation, plates with colonies of 30–300 were counted and the overall microbial population (N) was calculated by accounting for their respective dilution factor and size of the BI. The surviving fraction of spores was determined from the N/N_0_, where N_0_ is the population of the non-irradiated control. Enumeration was performed for a total of three times per BI strip.

### Modeling of bacterial activation and inactivation

In this study, different models were used to explain and predict the response of bacterial spores under X-ray irradiation. This response was classified into activation and inactivation, both of which described the bacteria’s state. By activated, we refer to a bacterial spore having the potential to undergo further development via germination and outgrowth into a fully functional vegetative bacterial cell. By inactivated, we refer to a bacterial spore that can no longer develop into a fully functional vegetative bacterium. This includes the possibility of a bacterial spore germinating but not undergoing outgrowth. For inactivation, we also refer to vegetative bacterium has lost its potential to undergo outgrowth.

The studied models include: (i) first-order kinetics model (ii) Shull model (iii) Sapru model and (iv) probabilistic model. Analysis was made on the averaged (from triplicate) bacterial population. An interesting hike at low doses was observed; this was contrary to the typical population decrease described by other inactivation models such as the most conventional first-order kinetics:

$$\log\;\left(\frac{N(t)}{N_{0}}\right)=-k_{d}t$$ (1)

Here, only inactivation is considered, as indicated by its corresponding rate term, *k*_*d*_. Thus, modeling the inactivation kinetics of bacterial spores alone would not suffice, and an activation aspect would have to be considered. However, this is included in the Shull model [25]:

$$\log\;\left(\frac{N(t)}{N_{0}}\right)=\log\;\left(\frac{k_{a}}{k_{a}-k_{d}}((1-e^{-(k_{a}-k_{d})t})e^{-k_{d}t})\right)$$ (2)

Here, both activation and inactivation are considered, as represented by *k*_*a*_ and *k*_*d*_ respectively. In the Shull model, inactivation of spores requires that the spores be first activated. This was further refined by in the Sapru model [26]:

$$\log\;\left(\frac{N(t)}{N_{0}}\right)=\log\;\left(\frac{k_{a}}{k_{a}+k_{d1}-k_{d2}}((1-e^{-(k_{a}+k_{d1}-k_{d2})t})e^{-k_{d2}t})\right)$$ (3)

Here, the two paths of inactivation are distinguished–directly from the spore (by *k*_*d1*_) and after the activation of the spore (by *k*_*d2*_). It should be noted that the Shull and Sapru models have been simplified here, with the initial population (*N*_*0*_) comprising only spores. This is based on the prior knowledge that our BI strips contain no activated spores.

An alternative to the above mentioned kinetic models is a probabilistic model reported by Horowitz *et al*. [27]. This model offers a simplification to the complex and multiple pathways a spore can undergo before deactivation. It accounts for (i) the collection of events that lead to inactivation and (ii) the collection of events that lead to activation and division. Each collection of events can be described by probability functions, as follows:

$$P_{a}(t)=\frac{p_{a}}{1+\exp(k_{a}(\tau_{a}-t))}$$ (4)

$$P_{d}(t)=\frac{p_{d}}{1+exp(k_{d}(\tau_{d}-t))}$$ (5)

Each equation is a sigmoid function describing the evolution of probabilities with time for both inactivation and activation. Each function eventually reaches a maximum probability of *p*. This happens following a delay of *τ* and an exponential increase at a rate of *k*. A probability function limit can then be written as:

$$g(t)=\int_{0}^{t}(P_{a}(s)-P_{d}(s))ds$$ (6)

Where the expected population is further given by:

$$N(t)=N_{0}\;\exp\left(g(t)\right)$$ (7)

$$\log\;\left(\frac{N(t)}{N_{0}}\right)=\log\;(exp(g(t)))$$ (8)

These four models were thus used in the curve fitting of experimental data. These fits were used in the determination of D_10_, D_37,_ 6- and 12-log reduction dose values. From the fits, curve parameters were also calculated to provide a quantitative measure of the different responses for the differently irradiated bacterial spores. This was done using the following equation [28]:

$$\log\;\left(\frac{N}{N_{0}}\right)=-ICt+n$$ (9)

Where *IC* is the inactivation constant–negative slope of the curve, and *n* is the extrapolation number–value of log*(N/N*_*0*_*)* at *t* = 0. The threshold dose (*D*_*q*_) can further be obtained at log*(N/N*_*0*_*)* = 0.

The fittings were also compared using the goodness of fit (R^2^) and the Akaike Information Criterion (AIC) [29, 30] values. The AIC penalizes models with higher number of parameters, and the model with the smallest AIC value for a particular set of data is preferred [31]. The AIC value for each model is calculated as follows [32]:

$$AIC_{i}=n+n\log2\pi +n\log\frac{{RSS}_{i}}{n}+2(K_{i}+1)$$ (10)

Here, *n* is the number of data points in the data set; *i* represents the different models; *RSS*_*i*_ is the residual sum of squares for a particular model; *K*_*i*_ is the number of parameters used in each model.

Although the AIC value provides a measure between the models, it does not clearly show the quantitative performance. Thus it is difficult to compare the model with the lowest AIC value to the one with the second lowest AIC value. Hence, the AIC values have to be normalized to reflect each model’s relative performance [33].

First, the difference in AIC of each model is computed with respect to the model with the lowest AIC value:

$$\Delta AIC_{i}=AIC_{i}-minAIC$$ (11)

Then, the difference is transformed to the likelihood values, and then normalized to the sum of all the likelihood values to obtain their respective Akaike weight such that the sum of the Akaike weights for all the models being evaluated is unity:

$$w_{i}=\frac{exp(-0.5\times \Delta AIC_{i})}{\sum exp(-0.5\times \Delta AIC_{i})}$$ (12)

## Results

### Inactivation of *Bacillus pumilus* spores by X-rays with different energies

**Fig 2:**
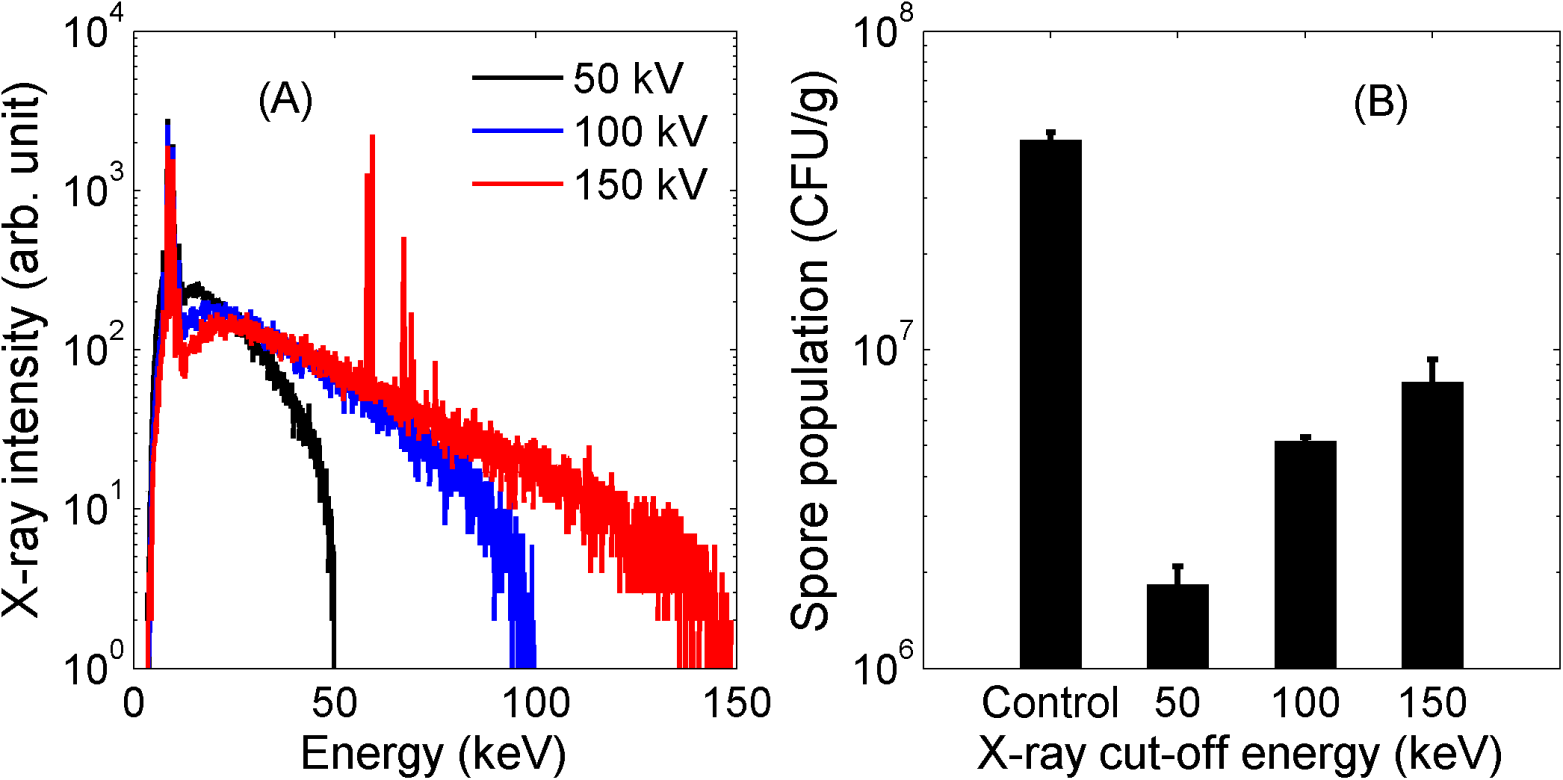
(A) Simulated X-ray spectrum at different tube voltages and (B) Inactivation of *Bacillus pumilus* spores under different X-ray spectrum (at a dose of 130 Gy).

*Bacillus pumilus* spores were exposed to three different X-ray irradiations with cut-off energies of 50, 100 and 150 keV. Their X-ray spectra were simulated using XENOS software (Field Precision LLC, USA) with consideration of the actual geometry of the X-ray source used in this study. These spectra are shown in the Fig 2A.

The different extents of inactivation are indicated by their corresponding remaining populations at a dose of 130 Gy, as shown in Fig 2B. Prior to irradiation, the population of *Bacillus pumilus* in the BI strip was measured to be (4.55 ± 0.25) x 10^7^ CFU.g^-1^. A maximum 3-fold reduction was observed for the cut-off energy of 50 keV, while only 2-fold and 1-fold reductions were seen for the X-ray spectra with 100 and 150 keV cut-off energies, correspondingly. It should be noted that at 195 Gy, the limit of detection for the total bacterial count was reached for the BI strip exposed to X-ray irradiation with the cut-off energy of 50 keV. At 260 Gy, the limit of detection was reached for the other two cut-off energies. Hence, analyses of results were only made to 130 Gy.

### Comparison of different models

**Fig 3:**
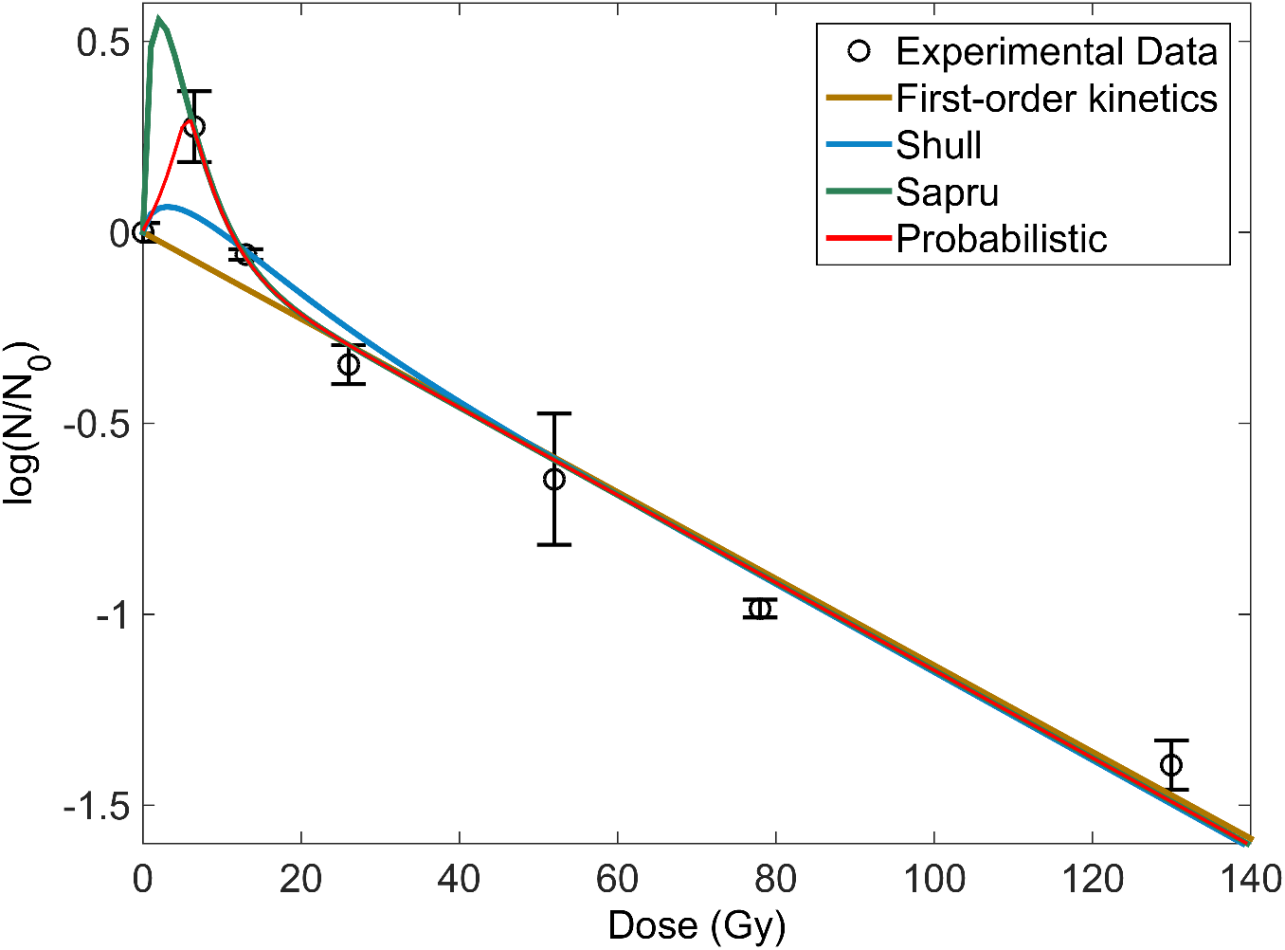
Comparison of fit for first-order kinetics model, Shull model, Sapru model and Probabilistic model. Irradiation was conducted on *Bacillus pumilus* spores with X-ray cut-off energy of 50keV.

In general, the Sapru and probabilistic model had comparable and the best goodness of fit as shown in Fig 3 and Table 1. The probabilistic model was computed to have lowest AIC values and highest Akaike weight values as shown in Tables 2 and 3, correspondingly.

**Table 1:** Goodness of fit (R^2^) values for the four models based on averaged data.

X-ray cut-off energy (keV)	Models			
	First-order	Shull	Sapru	Probabilistic
50	0.918±0.033	0.950±0.032	0.979±0.023	0.979±0.023
100	0.815±0.051	0.922±0.018	0.969±0.013	0.965±0.013
150	0.659±0.024	0.885±0.010	0.959±0.005	0.951±0.018

**Table 2:** Calculated AIC values for the four models based on averaged data.

X-ray cut-off energy (keV)	Models			
	First-order	Shull	Sapru	Probabilistic
50	8.72±3.16	6.58±5.57	0.06±7.80	-5.59±7.84
100	11.23±2.28	7.00±1.94	2.38±3.56	-2.45±2.81
150	12.82±1.00	6.95±0.52	1.51±1.76	-2.35±3.43

**Table 3:** Calculated Akaike weight values (in percentage) for the four models based on averaged data.

X-ray cut-off energy (keV)	Models			
	First-order	Shull	Sapru	Probabilistic
50	0.07±1.18%	0.22±1.24%	5.60±0.14%	94.12±2.35%
100	0.10±0.20%	0.81±1.25%	8.13±3.56%	90.97±2.28%
150	0.04±0.05%	0.83±1.07%	12.55±6.58%	86.57±7.69%

**Fig 4:**
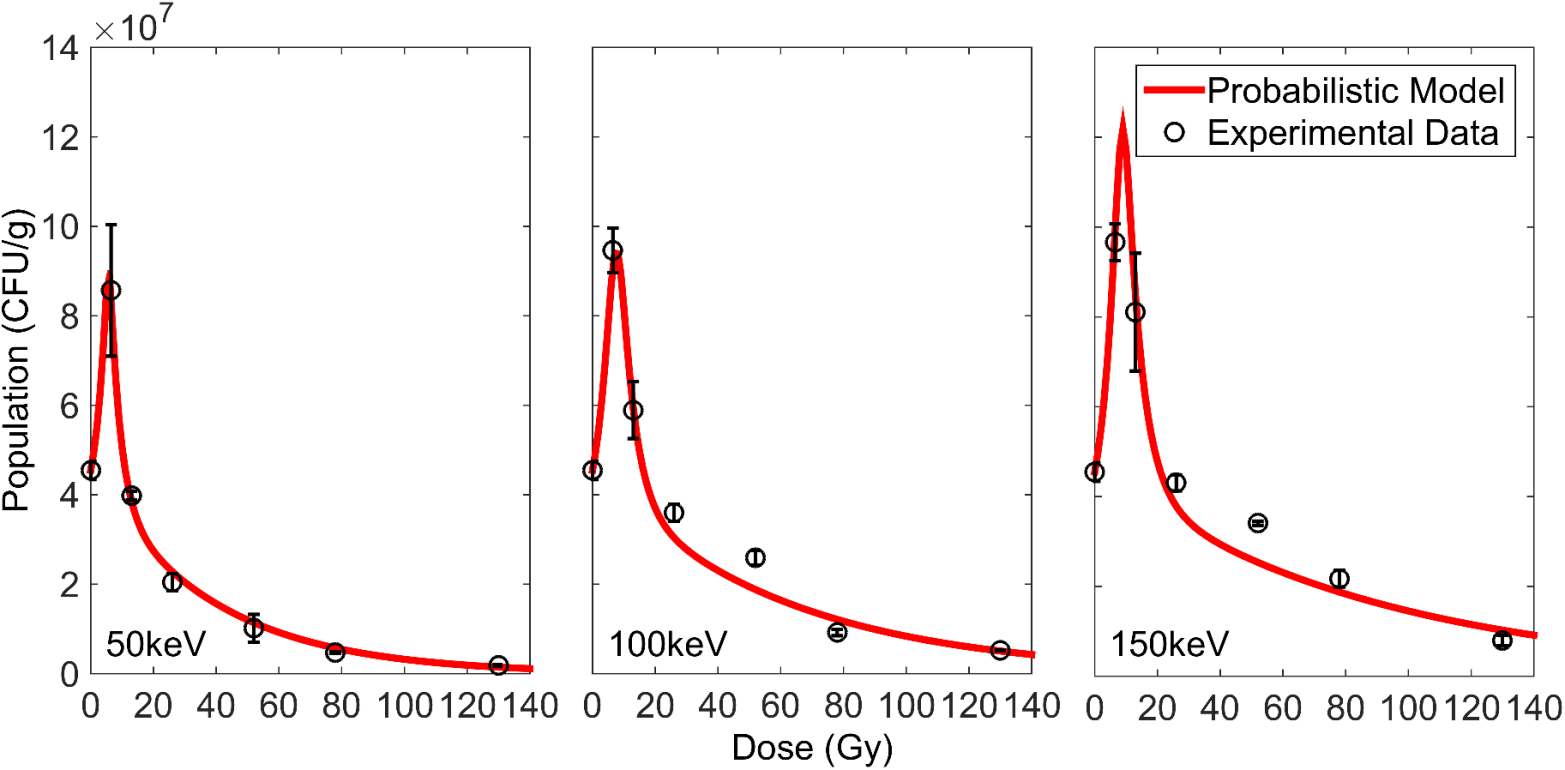
Activation and inactivation curves of *Bacillus pumilus* spores irradiated by X-rays of different cut-off energies. Fitted curves were obtained from the probabilistic model.

Fig 4 shows the dose effect of X-ray irradiation on *Bacillus pumilus* spores under the three different cut-off energies. Interestingly, at low doses of irradiation (below 13 Gy), an increase in the bacterial population was observed. This was followed by a decrease at higher doses. The same response was observed for all three cut-off energies.

Based on the fittings, the D_10_, D_37_, 6- and 12-log reduction doses were calculated for all cut-off energies. These computed values are summarized in Table 4. From the fitted curves, curve parameters were obtained for the first instance of inactivation (negative slope). These values indicate inactivation and are summarized in Table 5.

**Table 4:** Calculated D_10_, D_37_, 6- and 12-log reduction dose values (Gy) for different X-ray cut-off energies for the four models.

X-ray cut-off energy (keV)	Models				COV*
	First-order	Shull	Sapru	Probabilistic	
**50**					
D_37_	38±1	39±1	38±1	38±1	1.1%
D_10_	88±2	87±1	87±2	87±2	0.8%
6-log	530±9^#^	521±7	523±9	524±8	0.7%
12-log	1059±18^#^	1043±16	1047±17	1047±17	0.7%
**100**					
D_37_	61±3	64±5^#^	59±3	59±3	3.8%
D_10_	141±8	129±3	135±6^#^	137±6	3.7%
6-log	847±48^#^	766±10	813±33	821±38	4.1%
12-log	1695±97^#^	1532±20	1630±66.4	1640±77.0	4.2%
**150**					
D_37_	92±6^#^	90±2	85±5	86±4	3.8%
D_10_	211±12^#^	156±9^#^	191±16	198±9	12.5%
6-log	1268±75^#^	830±110^#^	1148±93	1190±54	17.4%
12-log	2538±149^#^	1659±220^#^	2298±185	2380±108	17.4%

*COV: Coefficient of variation (= standard deviation/mean) of data.

^#^ indicate reduction dose values that are significantly different (P ≤ 0.05 based on a two-tailed paired t-test) from the probabilistic model.

**Table 5:** Curve characteristics for different X-ray cut-off energies for the four models.

X-ray cut-off energy (keV)	Models			
	First-order	Shull	Sapru	Probabilistic
**50**				
IC (Gy^-1^)	0.026±0.0004^#^	0.026±0.002^#^	0.154±0.065	0.162±0.007
n	-	0.27±0.01^#^	1.66±0.62	1.82±0.21
D_q_ (Gy)	-	10±1	11±1	11±1
**100**				
IC (Gy^-1^)	0.016±0.001^#^	0.015±0.002^#^	0.057±0.008^#^	0.115±0.011
n	-	0.31±0.03^#^	1.07±0.08^#^	1.81±0.14
D_q_ (Gy)	-	21±4^#^	19±4	16±2
**150**				
IC (Gy^-1^)	0.011±0.001^#^	0.010±0.005^#^	0.039±0.010	0.077±0.019
n	-	0.39±0.04^#^	1.06±0.06	1.59±0.17
D_q_ (Gy)	-	39±3^#^	28±6	21±4

^#^ indicate reduction dose values that are significantly different (P ≤ 0.05 based on a two-tailed paired t-test) from the probabilistic model.

## Discussion

At a dose of 130 Gy, results in Fig 2B show that lower energy X-rays are more effective in inactivating bacterial spores than higher energy X-rays. This can be attributed to their differences in linear energy transfer (LET). LET is briefly defined as the average energy imparted over a distance by a charged particle to the medium in which it is traversing. Although by definition LET is limited to charged particles, it has become commonly associated with X-rays and gamma rays. This is because under these irradiations energy transfer still can occur via electron tracks. Typically, laboratory X-rays with cut-off energies of 50 keV or more are referred to as low LET radiations. [18] For the X-rays used in this work, we estimate their LET to be 1.7, 1.0 and 0.7 keV.μm^-1^ (calculated using ESTAR program of NIST [34]) for the cut-off energies of 50, 100 and 150 keV respectively. At low LET irradiations, it has been reported that higher LET results in increased relative biological effect (RBE) [19, 20]. This translates to more efficient inactivation, as observed in the results for lower energy X-ray irradiation. It should also be noted that RBE generally increases with LET until it crosses into high LET radiations (eg. by α-particles). Here, the likelihood of multiple “wasted hits” increases and therefore RBE decreases.

The observation of lower energy X-ray irradiation being more effective has also been made in previously reported research, but on different cell type [15, 35]. From the application point of view, these highlight the benefits of low-energy X-rays in terms of sterilization efficacy; ease of generation and less stringent shielding requirements. Low-energy X-rays therefore become a promising new irradiation method, especially for in-line sterilization of thin samples where high penetration is not required.

On the other hand, irradiation at low doses results in a bacterial population increase corresponding to a form of bacterial spore activation. Previous studies have suggested that a major effect of ionizing radiation is the disruption of cell membranes [36]. Alterations in membrane permeability could thus enable the influx of nutrients required for spore activation. However, further study is needed in order to clearly understand the detailed mechanism of radiation-induced activation of spores in the kiloelectron volt X-ray regime. For food irradiation in particular, it becomes important to note that the product will need to be sufficiently irradiated in order to avoid the potential activation of any present bacterial spores.

The extent of activation at low doses was presented as an approximate two-fold increase in bacterial population. A closer observation of plots in Fig 4 further reveals an increased extent of activation with increasing X-ray energies. At higher doses, the bacterial population was observed to eventually decrease. The rate of decrease was observed to be slower as the cut-off X-ray energy increased. This indicated a higher effectiveness for irradiation under lower X-ray energies.

Comparison of the different models via R^2^ and AIC analyses showed that the probabilistic model performed the best. Based on the R^2^ analysis, the probabilistic and Sapru models were fairly comparable. Although this could be an unfair comparison due to the larger number of coefficients present in the former, the further conduct of AIC (where both goodness of fit and parsimony can be accounted for) indicated otherwise. Despite of this, we suggest that the Sapru model be applied to fit such activation-inactivation population curves. This is because it is simpler and more straightforward to associate to the assumed pathways experienced by the bacterial spores. On the other hand, if a multitude of pathways were expected, fitting with the probabilistic model would be simpler. It was also noted that the fitted curves for Sapru and probabilistic models in Fig 3 very much differed before the first data point at 13 Gy. More data points should thus be obtained to better compare the fits. This was not done in this work due to equipment limitations.

Examining the D_10_, D_37_, 6- and 12-log reduction dose values in Table 4, it is noted that the doses (required to obtain the respective bacterial population decrements) increases with the X-ray cut-off energies. This corresponds to the earlier discussion on lower energy X-rays being more efficient in bacterial inactivation. Comparing across the models, it is interesting to note that the different models agree (i.e. has a smaller COV) under specific criteria. First, deviations in the calculated dose values were lowest for X-ray irradiations of lower energies. This is reflected by the smaller COV values for calculated doses at the lowest X-ray cut-off energy of 50 keV. Since all four models are capable of accounting for inactivation, they naturally become more agreeable when inactivation dominates under lower energy X-ray irradiation, as mentioned earlier. This implies that for low-energy X-ray irradiations, prediction of doses can be made with any of the four models. Conversely, it was noted that the deviation of calculated doses became larger (i.e. has a larger COV) when activation became prominent under irradiation by higher energy X-rays. The Shull model was observed to overestimate the extent of inactivation, by predicting very much lower dose requirements. This is especially so when it was extrapolated to predict the low remaining bacterial population under D_10_, 6- and 12-log reduction doses. This is similarly observed in Fig 3, where the Shull model’s fit lies severely lower than the experimental data. Notably, the first-order kinetics model was able to predict doses closer to the better fitting models–Sapru and probabilistic models. This implies that for the prediction of doses required for low remaining bacterial population the simplest first-order kinetics model would provide a good estimate. However, as it is an overestimate of required doses, it would be less ideal for the optimization of sterilization processes. Relative to the best fitting probabilistic model, however, required doses for the first-order kinetics and Shull model were shown by the t-test to be significantly different. This was more apparent at higher reduction doses and for irradiation by X-rays of higher energies.

The curve characteristics, summarized in Table 5, offer a means of quantification for activation and inactivation. First, *IC* measures the rate of inactivation and corresponds to the negative slope of the population curve. The larger the value of *IC* hence indicates a more rapid decrease in bacterial population per Gy of X-ray. Comparison across the different cut-off energies shows that *IC* is the highest for the cut-off energy of 50 keV and the lowest for the cut-off energy of 150 keV. This provides a quantity to the earlier discussed inactivation efficiency and supports that low-energy X-rays are more efficient in inactivation. Next, *D*_*q*_ represents the threshold dose, when *N* = *N*_*0*_. When this dose is exceeded, when N<N_0_, the bacterial population essentially starts falling below its initial. *D*_*q*_ thus indicates the minimum dose required to start a sterilization process. As would be expected of the better inactivation efficiency of low-energy X-rays, *D*_*q*_ is smallest for the cut-off energy of 50 keV and largest for the cut-off energy of 150 keV. The above-discussed trends for *IC* and *D*_*q*_ are the same for all models, with the exception of the first-order kinetics model for *D*_*q*_ that always crosses zero. However, when examining *n* this is less true, with only *n* values consistently increasing in the Shull and probabilistic models. Since *n* represents the extrapolated value at zero dose, it is indicative of the extent of bacterial spore activation. A larger *n* simply implies more spore activation, as was observed with increasing X-ray cut-off energies. This also supports the earlier discussion on increased extents of activation when irradiated by X-rays of higher energies. Comparing between the models, it can be observed that the first-order kinetics and Shull models underestimate inactivation, mainly as their fitted curves fall below actual the data points. With regards to their ability to quantify activation, the Shull model is a definite underestimate. It is however unclear at this point whether the Sapru or probabilistic model offers a better fitting for the dose region for bacterial activation due to the limited data points.

In this study, it is also important to note that we evaluated the bacterial spores’ response to X-ray irradiation based only on the total viable bacterial count. Therefore, several factors pertaining to the bacterial spores’ specific states post-irradiation were not considered in this work. This includes the spores’ super-dormancy [37–39], germination and outgrowth heterogeneity [40, 41] as well as DNA repair [8, 42, 43]. These factors are typically affected by the spores’ environmental and growing conditions, which include the contents of the nutrient medium. For example, it has been shown that high salinity (sodium chloride content) in the nutrient medium causes diverse inhibitory effects on *Bacillus subtilis* spore germination [44]; similarly, heat treatments and incubation temperatures have also been reported to largely affect germination [45]. Although analysis of these factors was not made in this study, unbiased and consistent environmental and growing conditions were maintained for all experiments. This included verifying that salinity of the nutrient medium was below the threshold for germination inhibition, and that there was no presence of inhibitory agents that might prevent the germination of sub-lethally-damaged spores or outgrowth. Likewise, no agents that aided germination or outgrowth were present. In that regard, we assume in this study an outgrowth efficiency of 100%. Therefore, a safety (or correction) factor would need to be applied to the computed doses during the sterilization process design and validation so as to avoid an under estimation. However, it should be highlighted that this would not affect the relative comparisons made in this study, which focuses on evaluating the effectiveness of different X-ray energies. We do nevertheless acknowledge that future work, particularly in the sterilization process validation, should include reported techniques for determination of heterogeneity as well as spore germination and outgrowth efficiency [46–49]. This consists of methods such as time-lapsed phase contrast microscopy combined with fluorescence microscopy, Raman spectroscopy and the use of optical tweezers.

In summary, this study reports the response of endospore-forming bacteria *Bacillus pumilus* under different low-energy X-ray beam qualities. Results showed that this response comprised a combination of bacterial spore activation and inactivation processes. We reported an X-ray-induced activation of *Bacillus pumilus* spores at very low doses (below 13 Gy), which is followed by inactivation at higher doses. In this study, we also demonstrated that X-rays with lower energies provided better bacterial inactivation efficacy. We attributed this to the higher LET of lower energy X-rays. For X-ray irradiation with 50 keV cut-off, the dose needed for 12-log bacterial population reduction was calculated to be about 1000 Gy, which is about one order lesser in magnitude compared to gamma irradiation. From our R^2^ and AIC analyses of the four kinetic models, we concluded that the Sapru and probabilistic models were most precise in predicting bacterial spore activation and inactivation behavior.
